# The highly pathogenic H5N1 influenza A virus down‐regulated several cellular MicroRNAs which target viral genome

**DOI:** 10.1111/jcmm.13219

**Published:** 2017-06-13

**Authors:** Rong Wang, Ying‐Ying Zhang, Jian‐Sheng Lu, Bing‐Hui Xia, Zhi‐Xin Yang, Xu‐Dong Zhu, Xiao‐Wei Zhou, Pei‐Tang Huang

**Affiliations:** ^1^ Laboratory of Protein Engineering Beijing Institute of Biotechnology Beijing China; ^2^ The General Hospital of the PLA Rocket Force Beijing China

**Keywords:** H5N1 HPAI, H1N1, virus replication, MicroRNAs, host defence

## Abstract

Higher and prolonged viral replication is critical for the increased pathogenesis of the highly pathogenic avian influenza (HPAI) subtype of H5N1 influenza A virus (IAV) over the lowly pathogenic H1N1 IAV strain. Recent studies highlighted the considerable roles of cellular miRNAs in host defence against viral infection. In this report, using a 3′UTR reporter system, we identified several putative miRNA target sites buried in the H5N1 virus genome. We found two miRNAs, miR‐584‐5p and miR‐1249, that matched with the PB2 binding sequence. Moreover, we showed that these miRNAs dramatically down‐regulated PB2 expression, and inhibited replication of H5N1 and H1N1 IAVs in A549 cells. Intriguingly, these miRNAs expression was differently regulated in A549 cells infected with the H5N1 and H1N1 viruses. Furthermore, transfection of miR‐1249 inhibitor enhanced the PB2 expression and promoted the replication of H5N1 and H1N1 IAVs. These results suggest that H5N1 virus may have evolved a mechanism to escape host‐mediated inhibition of viral replication through down‐regulation of cellular miRNAs, which target its viral genome.

## Introduction

Avian influenza H5N1 virus continues to be zoonotically transmitted to humans, causing severe disease and posing a pandemic threat to public health 1. In patients with the HPAI subtype of H5N1 IAV, a rapid progression of primary viral pneumonia often occurs leading to multiple organ failure and death 2. The severe and often fatal disease in humans caused by the HPAI H5N1 virus might arise from many different mechanisms, including a broader tissue tropism of the virus beyond the respiratory tract, compared to the human seasonal influenza (H1N1) viruses; however, one of the most crucial pathogenic factors is the high viral replication efficiency of the HPAI H5N1 virus 3. In addition, the threshold of viral replication was demonstrated to play an ultrasensitive mechanism of influenza‐induced inflammation in a strain‐dependent manner, where highly pathogenic influenza virus generally had a lower viral titre threshold to induce the cytokines storm and strong inflammation 4. Controlling the viral replication in the host is the key to achieving good clinical outcome in patients with HPAI H5N1 infection. Although the molecular mechanisms of the high viral replication efficiency of HPAI H5N1 virus are currently unclear, there is growing evidence that hosts use endogenous microRNAs (miRNAs, *i.e*. single‐stranded RNA molecules of about 22 nucleotides in length that suppress gene expression by binding semi‐complementary to target mRNAs to trigger mRNA cleavage or inhibit mRNA translation 5, 6, 7, 8, 9, 10) to regulate viral replication by post‐transcriptional regulation of viral genes. However, there were several conflicting results that cellular miRNAs could actually promote or inhibit viral replication. Some studies showed that hepatitis C RNA viral replication could be enhanced by miR‐122 11, 12, while other studies demonstrated that many viral replication levels were not elevated in miRNA or siRNA‐deficient 293T cells 13, or were even inhibited by cellular miRNAs. For example, miR‐28‐3p inhibits human T cell leukaemia virus type 1 by specifically targeting the genomic gag/pol viral mRNA 14, and miR‐548 g‐3p suppresses the proliferation of dengue virus subtypes 1–4 by targeting 5′UTR 15.

In the case of influenza viruses, cellular miRNAs could inhibit replication by directly targeting viral genomes. MiR‐323, miR‐491 and miR‐654 had been shown to inhibit viral replication in MDCK cells infected with H1N1 IAV by binding to the conserved region of the IAV PB1 gene 16. In addition, an atypical miRNA, miR‐2911, was shown to significantly inhibit H1N1, H5N1 and H7N9 IAV replication in mice by directly targeting the IAV PB2 and NS1 genes 17. Moreover, the incorporation of artificial miRNA target sites into H1N1 and H5N1 reassortants could significantly reduce viral replication in mice 18. In addition, miR‐485 was demonstrated to target both host (RIG‐I) and influenza virus transcripts (PB1) to regulate antiviral immunity and restrict viral replication 19. Thus, there is strong evidence that miRNAs can inhibit IAV propagation in host cells.

Not surprisingly, some viruses, including IAV, have evolved mechanisms that evade the inhibitory effect of host miRNAs. One of the most likely mechanisms of viral escape is the selective loss of miRNA target sites in IAV viral genomes due to selection of point mutations during viral replication. Indeed, the highly lethal mouse‐adapted H9N2 IAV has lost its miR‐1904 target sites, resulting in increased virus growth 20. Another viral escape mechanism may occur *via* the down‐regulation of cellular miRNAs that target the IAV genome. Using a miRNA microarray profiling approach, distinct cellular miRNA expression patterns were found in the lungs of mice infected with the highly pathogenic 1918 and pandemic H1N1 influenza virus, and the repression of miRNAs was found more pronounced in the highly pathogenic 1918 strain than in the lowly pathogenic H1N1 strain 21. Suppression of miRNA expression was also observed in macaques infected with HPAI H5N1 strain 22, and in A549 cells infected with the highly pathogenic H7N7 strain 23. In particular, the let‐7c miRNA, which reduced H1N1 virus replication by inhibiting the expression of the IAV M1 protein 24, was highly up‐regulated in A549 cells infected with H1N1 IAV 24but was down‐regulated in H7N7 IAV infected A549 cells 23. Moreover, the mouse let‐7c miRNA homologue (mmu‐let‐7c) was down‐regulated in mice lungs infected with the HPAI H5N1 virus 25. However, it remains unclear whether the high viral replication efficiency of the HPAI H5N1 virus in humans is related to viral suppression of cellular miRNA expression.

In this study, we aim to investigate whether the HPAI H5N1 virus hijacked the host cellular miRNAs targeting its viral genome. We used the HPAI H5N1 genome‐based luciferase reporter assay to determine the miRNAs that specifically target the HPAI H5N1 viral genome, and determine whether these miRNAs were down‐regulated after HPAI H5N1 infection. We identified two miRNAs (miR‐584‐5p and miR‐1249) targeting the HPAI H5N1 viral genome whose expression was notably down‐regulated in A549 cells infected with the HPAI H5N1 virus.

## Materials and methods

### Cell culture

293FT, A549 and MDCK cells were purchased from the cell culture centre of Institute of Basic Medical Sciences (IBMS, Beijing, China). These cell lines were maintained in Dulbecco's modification of Eagle's medium (DMEM) (Gibco, CA, USA) supplemented with 10% foetal bovine serum (FBS) (HyClone, UT, USA) at 37°C under 5% CO_2_ with 95% air atmosphere.

### Plasmid construction

In the 3′‐UTR reporter analysis experiments, pMIR‐REPORT (Ambion, TX, USA), which is the luciferase expression vector, was used as the parent vector. We used a PCR from A/goose/Jilin/hb/2003(H5N1) to amplify the 16 segments of the H5N1 IAV (the PB2, PB1, PA, HA, NP, NA, M and NS genes; both forwards and backwards, and the backwards were labelled as pMIR‐X‐R) and sub‐fragments of the PB2 gene (the PB2‐1, PB2‐2, PB2‐3, PB2‐4 and PB2‐5). These were then directionally cloned into the 3′‐UTR of the luciferase gene in the pMIR‐REPORT vector. PCR was used to amplify H1N1 PB2 (from A/Beijing/501/2009) and H5N1 PB2 for Western blot assays. To generate the c‐MYC‐PB2 (H1N1) and c‐MYC‐PB2 (H5N1), they were subsequently cloned into the pcDNA4.0 vector. For the purpose of determining the binding sites of the miRNA in the PB2 gene, nucleotide sequences of potential sites in the PB2 gene were mutated, using overlap extension PCR, in pMIR‐PB2 by generating pMIR‐PB2‐S/M series vectors (pMIR‐PB2‐S(584), pMIR‐PB2‐M(584), pMIR‐PB2‐S(1249), pMIR‐PB2‐M(1249); S: synonymous mutation, M: missense mutation).So that we could confirm the constancy of polymerase, we completely sequenced all inserts.

### Prediction of miRNA‐binding sites

The principles of miRNA target recognition 26, 27, 28, 29 were used to predict MiRNA binding sites. Briefly, Segal Lab of Computational Biology at http://genie.weizmann.ac.il/pubs/mir07/mir07_prediction.html was used to predict potential targets for miRNAs. RNAHybrid at http://bibiserv.techfak.uni-bielefeld.de/ and the RNA22 miRNA target predictor at http://cbcsrv.watson.ibm.com/rna22.html were used in order to corroborate selected miRNA‐target gene pairs.

### MiRNA inhibitors and expression vectors

MiRNA inhibitors were purchased from Ribobio (Guangzhou, China). The pCDH‐CMV‐MCS‐EF1‐copGFP (CD511B‐1) vectors (System Biosciences (SBI, Shanghai, China) were used to construct miRNA expression vectors according to the manufacturer's protocol. Gene sequences for miRNAs were acquired from the miRBase at http://www.mirbase.org/. The pre‐miRNA (60–70 nts) and 300–500 nts of flanking sequence were PCR amplified from human genomic DNA and subcloned into the miRNA expression plasmid. The resulting miRNA expression vectors were confirmed by sequencing.

### Virus infection

The A549 cells were infected with H1N1 IAV (A/Beijing/501/2009) or H5N1 AIV (A/goose/Jilin/hb/2003). Phosphate‐buffered saline (PBS) was used to wash the cells three times. Viral infection was performed in infection medium. This comprised culture medium, lacking FBS, but containing TPCK‐trypsin (1 μg/ml) (Promega, WI, USA) and BSA (1.5%). The virus MOIs were 1, 0.1, or 0.01, depending on the assay. The cells were incubated for 60 min. and then washed with PBS. Infection medium was then added. The infected cells were cultured in 5% CO_2_ at 35°C.

### Virus titres

The virus titres from culture supernatants of A549 cells were determined by measuring 50% Tissue Culture Infective Dose (TCID_50_) in MDCK cells. The titres were evaluated by the method described by Reed and Muench 30.

### Transfection

Lipofectamine 2000 transfection reagent (Invitrogen, CA, USA) was employed to transfect plasmids into cells. MiRNA inhibitors were transfected into cells using *Trans*IT‐TKO transfection reagent (TaKaRa, Dalian, China).

### Determination of miRNA binding sites

A549 cells were plated in 24‐well plates for the purpose of determining the viral segment with the candidate miRNA binding sites. Upon reaching confluence of 70–90%, the cells were cotransfected using the pMIR‐REPORT vector with the candidate viral gene (0.25 μg) and the pBIND‐control vector (0.05 μg). Six hours later, half of the duplicates were infected with H5N1 IAV at a Multiplicity of Infection (MOI) of 1, and half of the duplicates were infected as mock. The cells were then harvested 24 hrs for relative luciferase assay analysis.

### Determination of gene expression

To see whether miRNAs have a direct repression of candidate viral segment, 293 FT cells were seeded in 24‐well plates, and the 0.6 μg of miRNA expression vectors, 0.1 μg of pMIR‐REPORT vector containing the appropriate viral gene and 0.05 μg of the pBIND‐control vector were used to cotransfect the cells at a confluence of 70–90%. Forty hours later, harvesting of the cells took place for the purpose of relative luciferase assay analysis. The empty pMIR‐REPORT vector and pBIND‐control vector acted as negative controls.

For miRNA inhibitors, 200 nM miRNA inhibitor, 0.05 μg of pMIR‐REPORT vector containing H1N1 or H5N1 PB2 gene and 0.05 μg of the pBIND‐control vector were used to cotransfect the cells.

### Determination of protein expression

In order to establish whether miR‐584‐5p, and miR‐1249 were able to down‐regulate c‐MYC‐PB2 (H1N1 and H5N1) expression, 293FT cells were seeded in 24‐well plates. At a confluence of 70–90%, the cells were cotransfected with 0.2 μg of the appropriate miRNA expression vector and 0.6 μg of the appropriate c‐MYC‐PB2 expression vector. Thirty hours later, they were harvested for Western blot analysis.

### Determination of viral replication

A549 cells were plated in 24‐well plates for the purpose of establishing the effects of miRNAs on the transcript level of PB2 and NP genes and the replication of H5N1 and H1N1 IAV. On reaching a confluence of 70–90%, the cells were transfected with 0.8 μg of the miRNA expression vectors or 200 nM miRNA inhibitors. Twenty‐four hours later H5N1 or H1N1 IAV was used to infect the cells at a MOI of 0.1 or 0.01. Real‐time PCR was used to measure the transcript levels of the PB2 and NP genes. Western blot was used to detect the protein level of HA. TCID_50_ was used to determine viral replication.

### Luciferase assay

The dual‐luciferase reporter assay system kit (Promega, WI, USA) was used for the luciferase assays; the protocol recommended by the manufacturer was followed. In brief, cold PBS was used to wash harvested cells once. The passive lysis buffer (100 μl) was added to the cells. Centrifugation at 12,000 × *g* for 30 sec. was used to collect the supernatants at 10 min. The Spectra Max L (Molecular Devices, CA, USA) was used to determine the relative luciferase expression values. The relative luciferase expression equals the expression of firefly luciferase (pMIR‐REPORT) divided by the expression of Renilla luciferase (pBIND‐control vector).

### Real‐time PCR analysis

To detect PB2 and NP mRNA: Total RNA was prepared. Then, according to the manufacturer's instructions, the TransScript First‐Strand cDNA Synthesis SuperMix (Transgen, Beijing, China) was used to reverse transcribe 0.4 μg total RNA into cDNA. Oligo(dT) was used as primers. The cDNA was then used to measure the expression of PB2 and NP mRNA. The level of gapdh mRNA acted as a control.

To detect miRNAs: The cellular expression level of miRNAs was determined by preparing total RNA, and then reverse transcribing 0.4 μg of it into cDNA, by means of the TransScript First‐Strand cDNA Synthesis SuperMix (Transgen); the protocol suggested by the manufacturer was followed. Primers for cDNA synthesis and real‐time PCR were purchased from Ribobio. U6 RNA acted as a control.

TransStart Green qPCR SuperMix (Transgen) was used for real‐time PCRs. The cycling conditions were 95°C for 30 sec., then 40 cycles at 95°C for 10 sec. and at 60°C for 30 sec. After 1 min. at 95°C and 1 min. at 55°C, the melt curve was determined from 55°C to 95°C, with 10 sec. at each 0.5°C interval. The Bio‐RAD IQ5 detection system was used for real‐time PCR. Data were analysed using the 2^−▵▵Ct^ method 31.

### Statistical analysis

All the statistical analysis was carried out in GraphPad Prism 5 (GraphPad Software, CA, USA) with *t‐*test, and *P* value <0.05 was considered statistically significant.

## Results and Discussion

### The PB2 gene of the H5N1 IAV harbors potential miRNAs binding sites respond to H5N1 viral infection

**Figure 1:**
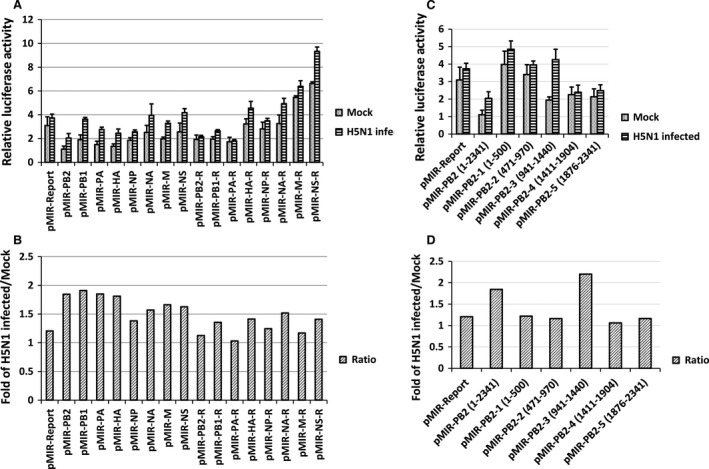
Potential miRNA binding sites on the PB2 gene that respond to H5N1 viral infection. (**A**,** C**) The relative luciferase activity from the pMIR‐REPORT constructs carrying one segment of the H5N1 IAV. A549 cells were plated in 24‐well plates. Upon reaching confluence of 70–90%, the cells were cotransfected using the pMIR‐REPORT series vectors (0.25 μg) and the pBIND‐control vector (0.05 μg). Six hours later, half of the duplicates were infected with H5N1 IAV at a MOI of 1, and half of the duplicates were infected as mock. The cells were then harvested 24 hrs for relative luciferase assay analysis. The experiments were carried out in duplicate and repeated twice. Data are expressed as mean ± S.D., *n* = 4. R: Reverse direction. (**B**,** D**) The changed fold of the relative luciferase activity after H5N1 IAV infected. For the same segment, the relative luciferase activity in cells infected with H5N1 was divided by the relative luciferase activity without infection, and then compared with the vector pMIR‐REPORT after the same calculation.

To determine whether host miRNAs could inhibit H5N1 IAV replication through binding to the viral genome, we used a H5N1 IAV genome‐based 3′‐UTR luciferase reporter assay. Eight segments of the H5N1 IAV genome were subcloned into the 3′‐UTR luciferase reporter plasmid (pMIR‐REPORT) in both the forward and reversed direction. The series of reporter plasmids were transfected into A549 cells with a pBIND‐control vector, and then half of the duplicates were infected with H5N1 IAV (MOI = 1), and the other half of the duplicates were infected as mock. After 24 hrs of infection, the cells were harvested for relative luciferase analysis. For each segment, the relative luciferase activity with H5N1 IAV infection was divided by mock, and then compared with the vector pMIR‐REPORT control. If the gene segment harbors potential miRNA binding sites that are down‐regulated by H5N1 IAV infection, the relative luciferase activity of H5N1 infection would be higher than the relative luciferase activity of mock, because of the decrease in the inhibitory effect of cellular miRNAs. As shown in Figure 1A, there was no notable change in the relative luciferase activity of the vector pMIR‐REPORT between the mock and H5N1 IAV infected. However, several viral gene segments showed a significant increase after H5N1 IAV infected. To compare the changes, the relative luciferase activity with H5N1 IAV infection was divided by mock, and then compared the ratio with the vector pMIR‐REPORT control (Fig. 1B). The ratio of PB2, PB1, PA and HA gene segments was more than 1.8‐fold in relative luciferase activity of H5N1 IAV infected compared to mock. This indicates that the PB2, PB1, PA and HA gene segments could harbor potential cellular miRNA binding sites.

To further determine the exact localization of these miRNA binding sites, five smaller fragments of the PB2 gene, and fragments of the PB1, PA, HA genes, were individually inserted into the 3′‐UTR of the luciferase gene in the pMIR‐REPORT vector. There were no statistical differences between the fragments of PB1, PA, HA (data not shown); however, the relative luciferase activity of the third fragment (941–1440 bp) of PB2 was substantially increased after H5N1 IAV infected (Fig. 1C). The ratio of PB2‐3 gene fragment was more than 2.0‐fold in relative luciferase activity of H5N1 IAV infected compared to mock (Fig. 1D). These results suggest that the PB2 gene harbors potential binding sites for miRNAs that were down‐regulated by H5N1 IAV.

### MiR‐584‐5p and miR‐1249 bind to conserved sites of PB2 gene

To determine the miRNAs that bind to the PB2‐3 gene fragment, we searched for putative miRNA binding sites in PB2‐3 gene fragment using the online miRNA prediction tool from the Segal Lab of Computational Biology, and confirmed the binding with the RNA22 and RNAHybrid programs. We identified six putative binding sites for miR‐584‐5p, miR‐1249, miR‐1237, miR‐328, miR‐346 and miR‐1248 (Table 1).

**Table 1:** The putative miRNA‐binding sites in the H5N1 PB2‐3 gene fragment

Gene	microRNA	Sites	Score
H5N1 PB2‐3	hsa‐miR‐584‐5p	1	−17.49
H5N1 PB2‐3	hsa‐miR‐1249	2	−16.13
H5N1 PB2‐3	hsa‐miR‐1237	2	−15.95
H5N1 PB2‐3	hsa‐miR‐328	3	−15.83
H5N1 PB2‐3	hsa‐miR‐346	1	−15.22
H5N1 PB2‐3	hsa‐miR‐1248	3	−15.08

**Figure 2:**
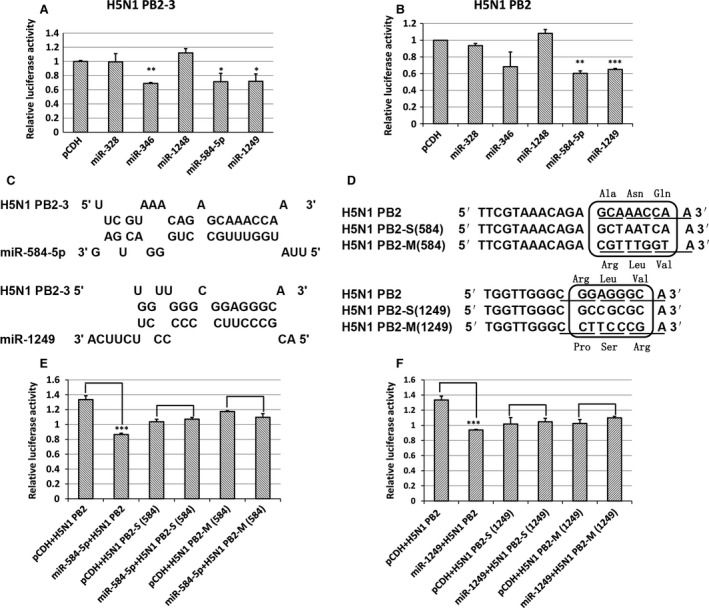
Confirmation of miR‐584‐5p and miR‐1249 binding sites on the IAV PB2 gene. (**A**,** B**) The relative luciferase activity of PB2‐3 or PB2 in 293FT cells overexpressing various miRNAs. The experiments were carried out in triplicate and repeated twice. Because the relative luciferase activity was different in every repeat, however the inhibition effect was in general the same, so the relative luciferase activity of the pMIR‐REPORT vector was set as 1 to compare conveniently. Data are expressed as mean ± S.D., *n* = 6. All statistical analyses were carried out (with pCDH as the control) using the *t*‐test. *P < 0.05, **P < 0.01, ***P < 0.001. (**C**) The model of hybridization between miR‐584‐5p or miR‐1249 and *pb2‐3* mRNA predicted using RNAHybrid software. (**D**) The synonymous (S) and missense mutant (M) nucleotide sequences of PB2 for the miR‐584‐5p and miR‐1249 binding sites. (**E**,** F**) The relative luciferase activity of PB2 in cells transfected with S/M mutant sequences of PB2 in 293FT cells overexpressing either miR‐584‐5p (**E**) or miR‐1249 (**F**) after infection with H5N1 IAV. The experiments were carried out in triplicate and repeated three times, and data are expressed as mean ± S.D., *n* = 3. All statistical analyses were carried out using the *t*‐test. ***P < 0.001.

Next, we determined whether these miRNAs inhibit expression of PB2‐3 by PCR‐amplifying the pre‐miRNA (60–70 nt) and its 300–500 nt flanking sequence from human genomic DNA and sub‐cloning it into the miRNA expression plasmid pCDH‐CMV‐MCS‐EF1‐copGFP(CD511B‐1). The pre‐miR‐1237 was unsuccessful to PCR‐amplify from human genomic DNA, so we couldn't construct the miR‐1237 expression vector. After cotransfection with the miRNA expression vectors, the pMIR‐PB2‐3 and the pBIND‐control vector, we analysed the relative luciferase activity in 293FT cells using the dual‐luciferase reporter assay system kit. As the relative luciferase activity was different in each repeat, and the inhibition effect was generally in the same, so we set the relative luciferase activity of the pMIR‐REPORT vector to 1 to facilitate comparison. Overexpression of miR‐584‐5p, miR‐1249, or miR‐346 significantly decreased the relative luciferase activity of cells transfected with PB2‐3 (Fig. 2A; *P* < 0.01). In addition, miR‐584‐5p and miR‐1249 also inhibited the expression of PB2 (Fig. 2B). These results indicate that the PB2 gene of H5N1 harbors the binding sites of miR‐584‐5p and miR‐1249.

RNAHybrid software was used to predict the modes of hybridization between the miRNAs and the PB2 mRNA and showed that the 5′ seed sequence of miR‐584‐5p and miR‐1249 perfectly matched a 7 or 8 nt sequence found in the PB2 gene (Fig. 2C). These predicted binding sites were then mutated through synonymous (S) or missense mutations (M) as shown in Figure 2D (The changes of AA were also shown in Fig. 2D.), and the pMIR‐PB2‐S/M vector series were generated. To analyse the effects of these mutations, 293FT cells were cotransfected with miRNA expression vectors, the pBIND‐control vector and either pMIR‐PB2‐S/M or pMIR‐PB2. The results showed that miRNA expression did not result in repression of the relative luciferase activity in cells transfected with pMIR‐PB2‐S/M (Fig. 2E and F). These results confirm that the PB2 gene harbors binding sites of miR‐584‐5p and miR‐1249, and that these binding sites are critical to the repression of reporter gene mediated by the two miRNAs targeting the PB2 gene.

It has been reported that the IAV can increase its replication efficiency through the loss of miRNA target sites *via* point mutations or changes to RNA secondary structures 32. Thus, the PB2 gene could be a potential hotspot for mutations during IAV evolution. To investigate this, we used a variety of influenza strains to perform the NCBI blast search for the miR‐584‐5p and miR‐1249 binding sites in the PB2 gene (Tables 2 and 3).

**Table 2:** The conserved binding sites of miR‐584‐5p in the PB2 gene compared across a selection of influenza viral strains^a^

Virus type and NCBI sequence no.	Sequence^b^
H1N1 KJ764739	AGTCCGAGGCGATCTGAATTTCGTAAACAGAGCAAACCAAAGATTAAACC
H1N2 KF013909	AGTCCGAGGCGATCTGAATTTCGTAAACAGAGCAAACCAAAGATTAAACC
H1N3 AB546177	AGTCCGAGGCGATCTGAATTTCGTAAACAGAGCAAACCAAAGATTAAACC
H3N1 KC986341	AGTCCGAGGCGATCTGAATTTCGTAAACAGAGCAAACCAAAGATTAAACC
H3N2 KM514352	AGTCCGAGGCGATCTGAATTTCGTAAACAGAGCAAACCAAAGATTAAACC
H4N3 JX454712	AGTCCGAGGCGATCTGAATTTCGTAAACAGAGCAAACCAAAGATTAAACC
H4N6 KF113552	AGTCCGAGGCGATCTGAATTTCGTAAACAGAGCAAACCAAAGATTAAACC
H5N1 CY094846	AGTCCGAGGCGATCTGAATTTCGTAAACAGAGCAAACCAAAGATTAAACC
H5N3 JQ737226	AGTCCGAGGCGATCTGAATTTCGTAAACAGAGCAAACCAAAGATTAAACC
H6N1 JF965054	AGTCCGAGGCGATCTGAATTTCGTAAACAGAGCAAACCAAAGATTAAACC
H6N6 KJ200716	AGTCCGAGGCGATCTGAATTTCGTAAACAGAGCAAACCAAAGATTAAACC
H7N7 KF260742	AGTCCGAGGCGATCTGAATTTCGTAAACAGAGCAAACCAAAGATTAAACC
H7N8 KC609818	AGTCCGAGGCGATCTGAATTTCGTAAACAGAGCAAACCAAAGATTAAACC
H9N2 JN852786	AGTCCGAGGCGATCTGAATTTCGTAAACAGAGCAAACCAAAGATTAAACC
H10N7 KC986344	AGTCCGAGGCGATCTGAATTTCGTAAACAGAGCAAACCAAAGATTAAACC
H10N9 KF260778	AGTCCGAGGCGATCTGAATTTCGTAAACAGAGCAAACCAAAGATTAAACC
H11N2 CY146737	AGTCCGAGGCGATCTGAATTTCGTAAACAGAGCAAACCAAAGATTAAACC
H11N9 CY146609	AGTCCGAGGCGATCTGAATTTCGTAAACAGAGCAAACCAAAGATTAAACC

a Collected from NCBI 21 October 2014.

b Underlined sequences indicate conserved miR‐584‐5p binding sites. Some influenza viruses are not shown in the table.

**Table 3:** The conserved binding sites of miR‐1249 in the PB2 gene compared across a selection of influenza viral strains^a^

Virus type and NCBI sequence no.	Sequence^b^
H1N1 CY115917	AGGGGTATGAGGAATTCACAATGGTTGGGAGGAGGGCAACAGCTATCCTGA
H1N2 KF013909	AGGGGTATGAGGAATTCACAATGGTTGGGCGGAGGGCAACAGCTATACTGA
H2N3 KC899708	AGGGGTATGAGGAATTCACAATGGTTGGGCGGAGGGCAACAGCTATTCTGA
H3N8 JN029578	AGGGGTATGAGGAATTCACAATGGTTGGGCGGAGGGCAACAGCTATTCTGA
H4N6 FJ349254	AGGGGTATGAGGAATTCACAATGGTTGGGCGGAGGGCAACAGCTATCCTGA
H5N1 HM006756	AGGGGTATGAGGAATTCACAATGGTTGGGCGGAGGGCAACAGCTATCCTGA
H5N3 EF597473	AGGGGTATGAGGAATTCACAATGGTTGGGCGGAGGGCAACAGCTATCCTGA
H6N1 KC785023	AGGGGTATGAGGAATTCACAATGGTTGGGCGGAGGGCAACAGCTATCCTGA
H6N6 HM804480	AGGGGTATGAGGAATTCACAATGGTTGGGCGGAGGGCAACAGCTATCCTGA
H7N1 CY005500	AGGGGTATGAGGAATTCACAATGGTTGGGCGGAGGGCAACAGCTATCCTGA
H7N9 KC609823	AGGGGTATGAGGAATTCACAATGGTTGGGCGGAGGGCAACAGCTATTCTGA
H9N2 CY024237	AGGGGTATGAGGAATTCACAATGGTTGGGCGGAGGGCAACAGCTATCCTGA
H9N2 EF154862	AGGGGTATGAGGAATTCACAATGGTTGGGCGGAGGGCAACAGCTATCCTGA
H11N9 CY146609	AGGGGTATGAGGAATTCACAATGGTTGGGCGGAGGGCAACAGCTATTCTGA
H13N2 JF965054	AAGGGTACGAGGAATTCACAATGGTTGGGCGGAGGGCAACAGCTATCCTGA

a Collected from NCBI 29 November 2016.

b Underlined sequences indicate conserved miR‐1249 binding sites. Some influenza viruses are not shown in the table. The mutations were shown in red colour.

By alignment of these influenza virus genomes, we found that the binding sites of the miRNAs were located in the coding region of the PB2 gene. We also found that both the miRNA seed sequences and viral RNA secondary structures in PB2 RNA were highly conserved across multiple influenza viral strains. This suggests that the HPAI H5N1 virus escape the inhibition of cellular miR‐584‐5p and miR‐1249 by direct regulation of miRNA expression, rather than loss of miRNA binding sites through point mutations or changes to RNA secondary structures.

### MiR‐584‐5p and miR‐1249 inhibited the replication of H1N1 and H5N1 IAV by down‐regulating PB2 expression in A549 cells

**Figure 3:**
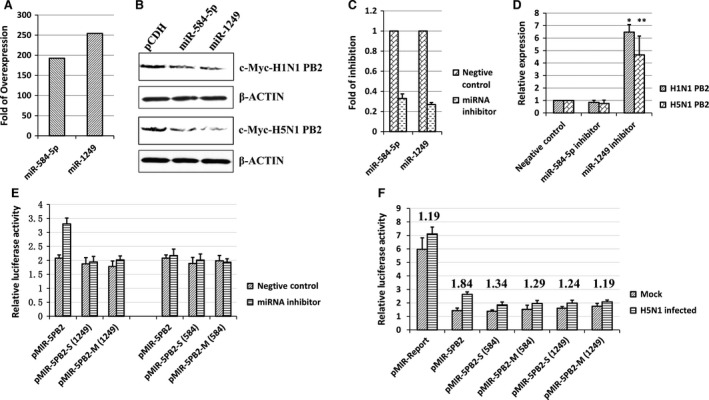
MiR‐584‐5p and miR‐1249 down‐regulate PB2 expression. (**A**) Relative expression of miR‐584‐5p and miR‐1249 after 48 hrs determined by real‐time PCR in A549 cells transfected with miRNA expression vector. (**B**) Western blot analysis of PB2 protein expression after H5N1 and H1N1 IAV infection in 293FT cells overexpressing miR‐584‐5p or miR‐1249 (pCDH vector as negative control). β‐actin protein expression was detected as a control. (**C**) Relative expression of miR‐584‐5p and miR‐1249 after 24 hrs determined by real‐time PCR in A549 cells transfected with 200 nM miRNA inhibitors. (**D**) The relative luciferase activity of PB2 with 200 nM miRNA inhibitors. (**E**) The relative luciferase activity of H5N1 PB2 mutants with 200 nM miRNA inhibitors. (**F**) The relative luciferase activity of H5N1 PB2 mutants after H5N1 IAV infection. The numbers mean the changed fold of the relative luciferase activity after H5N1 infection (H5N1 infected/mock). All experiments were repeated three times, and data are expressed as mean ± S.D., *n* = 3. All statistical analyses were carried out using the *t*‐test. *P < 0.05, **P < 0.01. Because the relative luciferase activity was different in every repeat, however the inhibition effect was in general the same, so the relative luciferase activity of the negative control was set as 1 to compare conveniently.

Our results showed that miR‐584‐5p and miR‐1249 specifically bind to highly conserved sites on the PB2 gene across a variety of IAV strains, indicating that miR‐584‐5p and miR‐1249 could regulate PB2 expression. Therefore, we subcloned H5N1 PB2 and H1N1 PB2 into the eukaryotic expression plasmid pcDNA 4.0 with a c‐MYC tag. As shown in Figure 3A, miR‐584‐5p and miR‐1249 overexpressed about 200‐fold more than the control in 293FT cells transfected with the miRNA expression plasmids. Overexpression of miR‐584‐5p or miR‐1249 resulted in lower expression of c‐MYC‐PB2 (H1N1) and c‐MYC‐PB2 (H5N1) in 293FT cells compared to cells without these miRNAs (*i.e*. the pCDH control) (Fig. 3B). Furthermore, we inhibited the miRNA expression in A549 cells using miRNA inhibitor by less than 40% (Fig. 3C). The inhibition of miR‐1249 significantly promoted the relative luciferase activity of H1N1 and H5N1 PB2 (Fig. 3D) without affecting the relative luciferase activity of H5N1 PB2 mutants (Fig. 3E). In addition, the H5N1 infection also didn't promote the relative luciferase activity of H5N1 PB2 mutants as well as H5N1 PB2 shown in Figures 1B and 3F. This suggested that miR‐584‐5p and miR‐1249 down‐regulated H1N1 and H5N1 IAV PB2 expression.

**Figure 4:**
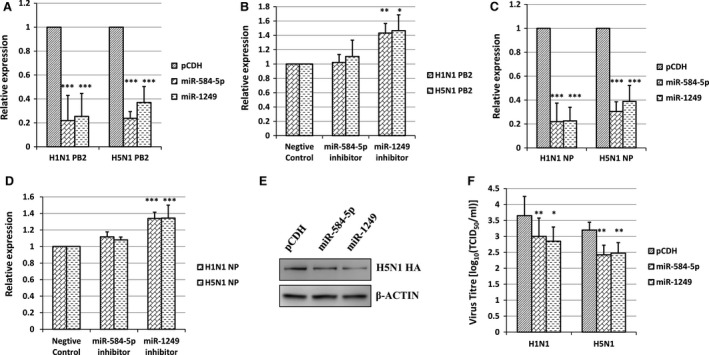
MiR‐584‐5p and miR‐1249 inhibit the replication of H5N1 and H1N1 IAV. (**A**) Relative expression levels of PB2 mRNA after H5N1 and H1N1 IAV infection in A549 cells overexpressing miR‐584‐5p or miR‐1249 (pCDH vector as negative control). (**B**) Relative expression levels of PB2 mRNA after H5N1 and H1N1 IAV infection in A549 cells inhibiting miR‐584‐5p or miR‐1249. (**C**) Relative expression levels of NP mRNA after H5N1 and H1N1 IAV infection in A549 cells overexpressing miR‐584‐5p or miR‐1249 (pCDH vector as negative control). (**D**) Relative expression levels of NP mRNA after H5N1 and H1N1 IAV infection in A549 cells inhibiting miR‐584‐5p or miR‐1249. (**E**) Western blot analysis of HA protein expression after H5N1 IAV infection in A549 cells overexpressing miR‐584‐5p or miR‐1249 (pCDH vector as negative control). β‐actin protein expression was detected as a control. (**F**) The virus titre (TCID_50_) after H5N1 and H1N1 IAV infection in the supernatant from A549 cells overexpressing miR‐584‐5p or miR‐1249 (pCDH vector as negative control). All experiments were repeated three times, and data are expressed as mean ± S.D., *n* = 3. All statistical analyses were carried out using the *t*‐test. *P < 0.05, **P < 0.01, ***P < 0.001. Because the relative expression was different in every repeat, however the inhibition effect was in general the same, so the relative expression of the control was set as 1 to compare conveniently in Figure 4A–D.

To further investigate the inhibitory effect of miR‐584‐5p and miR‐1249 during IAV infection, we examined the effect of miR‐584‐5p and miR‐1249 expression on H5N1 and H1N1 IAV replication in A549 cells. The A549 cells were transfected with the miR‐584‐5p and miR‐1249 expression vectors, and then infected with the H1N1 or H5N1 IAV (MOI = 0.1). After 60 hrs, the total RNA from the infected cells was collected to detect PB2 (and NP) gene expression by real‐time PCR. The results showed that the PB2 mRNA level was significantly reduced comparing to the negative control in cells overexpressing miR‐584‐5p or miR‐1249 (Fig 4A; *P* < 0.001). Meanwhile, transfection of miR‐1249 inhibitor significantly elevated the PB2 mRNA expression in A549 cells infected with H1N1 and H5N1 (Fig. 4B), indicating that miR‐1249 inhibited PB2 during IAV infection. The PB2 segment coding protein is a subunit of the influenza virus RNA polymerase and therefore contributes to viral transcription. In particular, the PB2 subunit binds to the cap structure at the 5′ end of host mRNA to generate short capped RNA fragments that are used as primers for viral transcription initiation 33, 34, 35. Therefore, inhibition of PB2 function can notably affect viral transcription and subsequent replication.

Given that the essential role of PB2 in viral transcription, the inhibition of PB2 expression by miR‐584‐5p or miR‐1249 is obligated to reduce the transcription of other IAV genes. Therefore, it is not surprising that NP transcription was found significantly reduced in H1N1 and H5N1 infected A549 cells overexpressing miRNAs (Fig. 4C; *P* < 0.001). In addition, NP transcription was up‐regulated in H1N1 and H5N1 infected A549 cells simultaneously with miR‐1249 inhibitor treatment (Fig. 4D; *P* < 0.001). As the expression of NP is usually considered as an indicator of IAV replication efficiency, these results indicate that overexpression of miR‐584‐5p and miR‐1249 can regulate the transcription of IAV genes that are critical for viral replication. Meanwhile, the protein level of H5N1 HA was significantly inhibited by miR‐584‐5p and miR‐1249 in H5N1 infected A549 cells (Fig. 4E). To determine the inhibitory effects of miR‐584‐5p and miR‐1249 on IAV replication, the viral titres (TCID_50_) of H5N1 and H1N1 IAV were monitored in the supernatants of A549 cells overexpressing miR‐584‐5p or miR‐1249. We found that overexpression of miR‐584‐5p and miR‐1249 significantly inhibited viral replication of H1N1 and H5N1 IAV with approximately equal inhibitory efficiency (Fig. 4F; *P* < 0.001). However, we found that inhibition of miR‐1249, but not miR‐584‐5p, could elevate the expression of PB2 and NP. This might be due to the non‐homogeneous expression level of miRNAs in A549 cells. Our unpublished miRNA chip data demonstrate that the miR‐1249 expression level was 80 times higher than that of miR‐584‐5p in A549 cells, although the expression level of both the miRNAs was reported no more than 0.1% of the total miRNA pool 36.

### H1N1 and H5N1 IAV differently regulate the expression of miR‐584‐5p and miR‐1249

**Figure 5:**
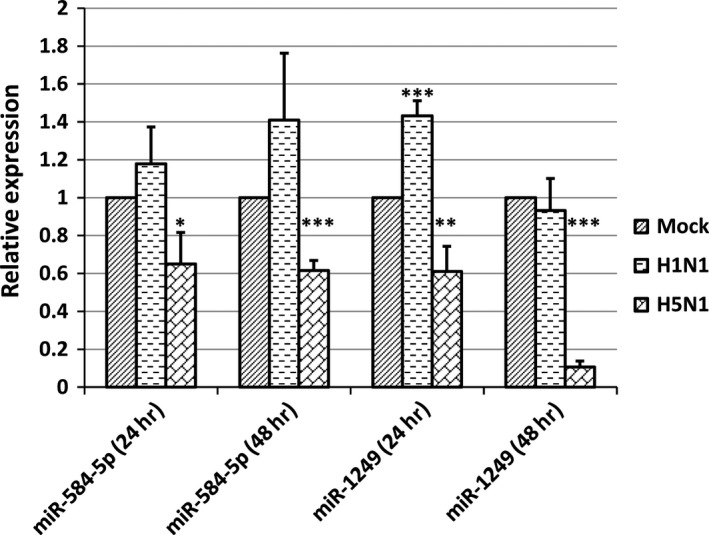
MiR‐584‐5p and miR‐1249 expression differs with H1N1 and H5N1 IAV infection. Relative expression of miR‐584‐5p and miR‐1249 after 24 hrs or 48 hrs determined by real‐time PCR in A549 cells infected with H1N1 or H5N1 IAV (MOI = 0.1). The experiments were repeated three times, and data are expressed as mean ± S.D., *n* = 3. All statistical analyses were carried out (with mock as the control) using the *t*‐test. *P < 0.05, **P < 0.01, ***P < 0.001. Because the relative expression was different in every repeat, however the inhibition effect was in general the same, so the relative expression of the control was set as 1 to compare conveniently.

HPAI H5N1 virus might escape the inhibitory effect of host defence through down‐regulation of certain cellular miRNAs that target its viral genome. Indeed, our results above indicated that miR‐584‐5p and miR‐1249 inhibited replication of H1N1 and H5N1 viruses in A549 cells. Therefore, we next determined whether H1N1 and H5N1 IAV infection could down‐regulate miR‐584‐5p and miR‐1249 expression. We infected A549 cells with H1N1 or H5N1 IAV (MOI = 0.1), and measured the expression of miR‐584‐5p and miR‐1249 using real‐time PCR after 24 hrs and 48 hrs. As shown in Figure 5, the expression of miR‐584‐5p and miR‐1249 were differently regulated by H1N1 and H5N1 IAV infection. Although miR‐584‐5p and miR‐1249 were down‐regulated by H5N1 infection 24 hrs and 48 hrs after infection, they were not down‐regulated by H1N1 IAV infection. Moreover, at some post‐infection time‐points, the expression of miR‐584‐5p and miR‐1249 was slightly up‐regulated by H1N1 IAV infection. This suggests that diverse strains of influenza virus can differently regulate the expression of miR‐584‐5p and miR‐1249, that is, highly pathogenic H5N1 IAV infection can down‐regulate miRNA expression, but lowly pathogenic H1N1 IAV cannot.

### H5N1 IAV has higher replication efficiency than H1N1 IAV

**Figure 6:**
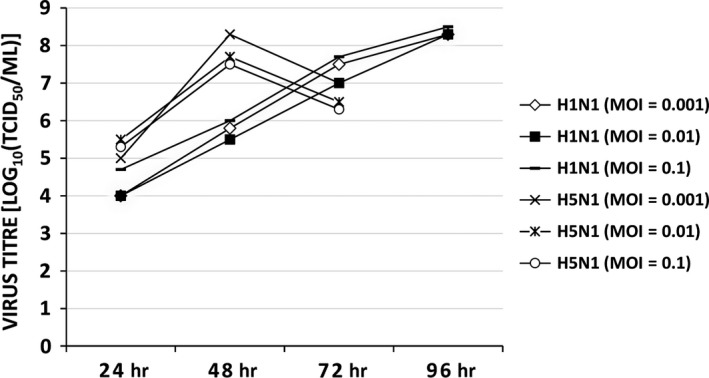
The replication of H5N1 and H1N1 IAV. Virus titre (TCID_50_) in MDCK cells infected with the H5N1 or H1N1 IAV (MOI at 0.001, 0.01 or 0.1), after 24, 48, 72 and 96 hrs.

Although miR‐584‐5p and miR‐1249 inhibited H5N1 and H1N1 IAV replication (Fig. 4F), the expression of miR‐584‐5p and miR‐1249 decreased only after H5N1 infection (Fig. 5), indicating that miRNA‐induced viral replication inhibition might be less efficient for H5N1 than H1N1 IAV. Indeed, as shown in Figure 6, the replication of H5N1 IAV reached its peak at 48 hrs post infection, much earlier than H1N1 IAV (96 hrs), indicating that H5N1 IAV had a higher replication efficiency. Nonetheless, H1N1 could achieve a higher viral titre than H5N1 after 96 hrs. However, the higher viral titres of H1N1 might be due to the fact that H5N1 IAV infection could cause cell death and shed off from cell culture plates at 48 hrs because of high level of replication and high pathogenicity. In conclusion, these results suggest that H5N1 IAV down‐regulation of miR‐584‐5p and miR‐1249 expression can explain why the H5N1 strain has higher replication efficiency and pathogenicity.

## Conclusions

Although cellular miRNAs are known to be critical for the antiviral responses in mammalian somatic cells, further understanding of the interactions between cellular miRNAs and IAVs are required. In this manuscript, we provided evidence that the HPAI H5N1 virus evolved a mechanism that inhibited the expression of cellular miR‐584‐5p and miR‐1249 to escape host antiviral responses and aid efficient viral replication. In particular, we identified a viral segment of H5N1 (PB2) that is regulated by cellular miRNAs, and identified two cellular miRNAs binding sites (miR‐584‐5p and miR‐1249) in PB2 coding region. We found that miR‐584‐5p and miR‐1249 were dramatically down‐regulated by H5N1 IAV, but not by H1N1 IAV infection. This differential miRNA expression pattern could reflect the specific viral escape mechanism of the HPAI H5N1 virus to evade the host miRNAs, thereby explaining the higher pathogenicity of H5N1 over H1N1. Furthermore, transfection with miR‐1249 inhibitor enhanced PB2 expression and replication of H5N1 and H1N1 IAVs. In conclusion, our findings improved our understanding of the interaction between the host defence and influenza A viral pathogenesis.

## Author contributions

PTH XWZ YYZ conceived and designed the experiments. RW BHX performed the experiments. RW JSL analysed the data. ZXY XDZ contributed reagents/materials/analysis tools. RW YYZ wrote the paper. All authors reviewed the manuscript.

## Conflict of interest

The authors confirm that there are no conflicts of interest.
